# Supplementary material for: An excitatory ventromedial hypothalamus to paraventricular thalamus circuit that suppresses food intake
Source: Nat Commun. 2020 Dec 10;11:6326. doi: 10.1038/s41467-020-20093-4 (PMC7728757; doi:10.1038/s41467-020-20093-4)
Supplement: Supplementary file 1 — Supplementary Information [file 41467_2020_20093_MOESM1_ESM.pdf]

Supplementary Information

An excitatory ventromedial hypothalamus to paraventricular thalamus circuit that suppresses food intake

Jia Zhang, Dan Chen, Patrick Sweeney, and Yunlei Yang*

Supplementary Figure 1

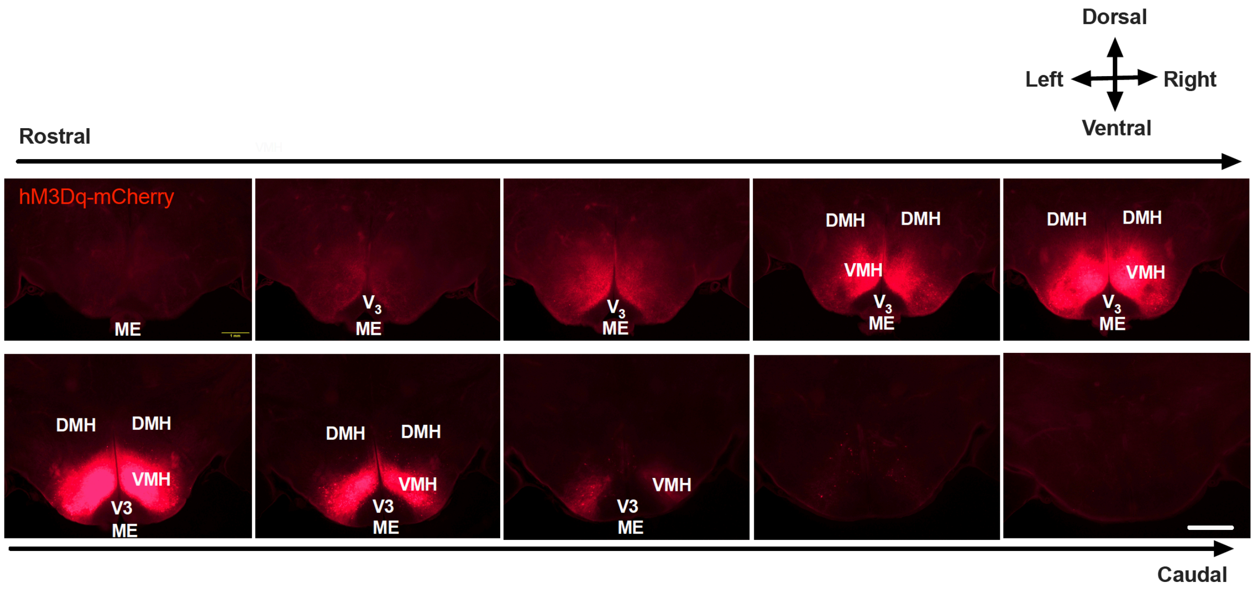

Supplementary Fig. 1. A series of confocal images of DREADD-transduced SF1 neurons. Representative sample images from rostral and caudal in a SF1-Cre mouse transfected with hM3Dq-mCherry in VMH SF1 neurons. Viral expressions were primarily observed in the center and dorsomedial VMH. Sparse infection was also observed in the ventral lateral area of VMH. Scale bar, 1 mm. ME, medium eminence; V_3, third ventricle; VMH, ventromedial hypothalamus.

Supplementary Figure 2

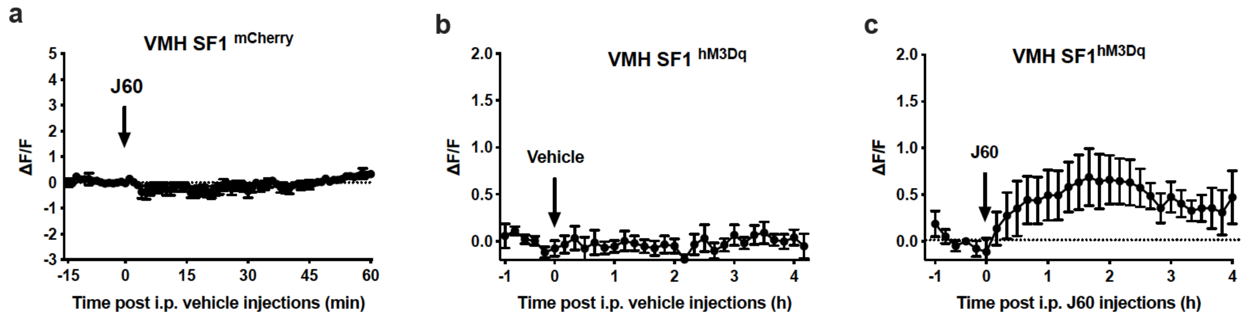

Supplementary Fig. 2. GCaMP$_{6f}$ signals recorded in SF1 neurons.

a, J60 administration via i.p. injections did not affect GCaMP$_{6f}$ signal in the SF1 mCherry-transduced mice (n=6). **b-c**, Four-hour real time monitoring of VMH SF1 neuron GCaMP$_{6f}$ signals in the SF1 hM3Dq-transduced mice treated with vehicle (**b**) or J60 (**c**) via i.p. injections (vehicle, n=6; J60, n=5). The GCaMP$_{6f}$ signals were presented at 1-min bins for (**a**) and 10-min bins for (**b**, **c**) respectively, and subtracted from the average basal ΔF/F for each experiment. Data represent mean ± s.e.m.

Supplementary Figure 3

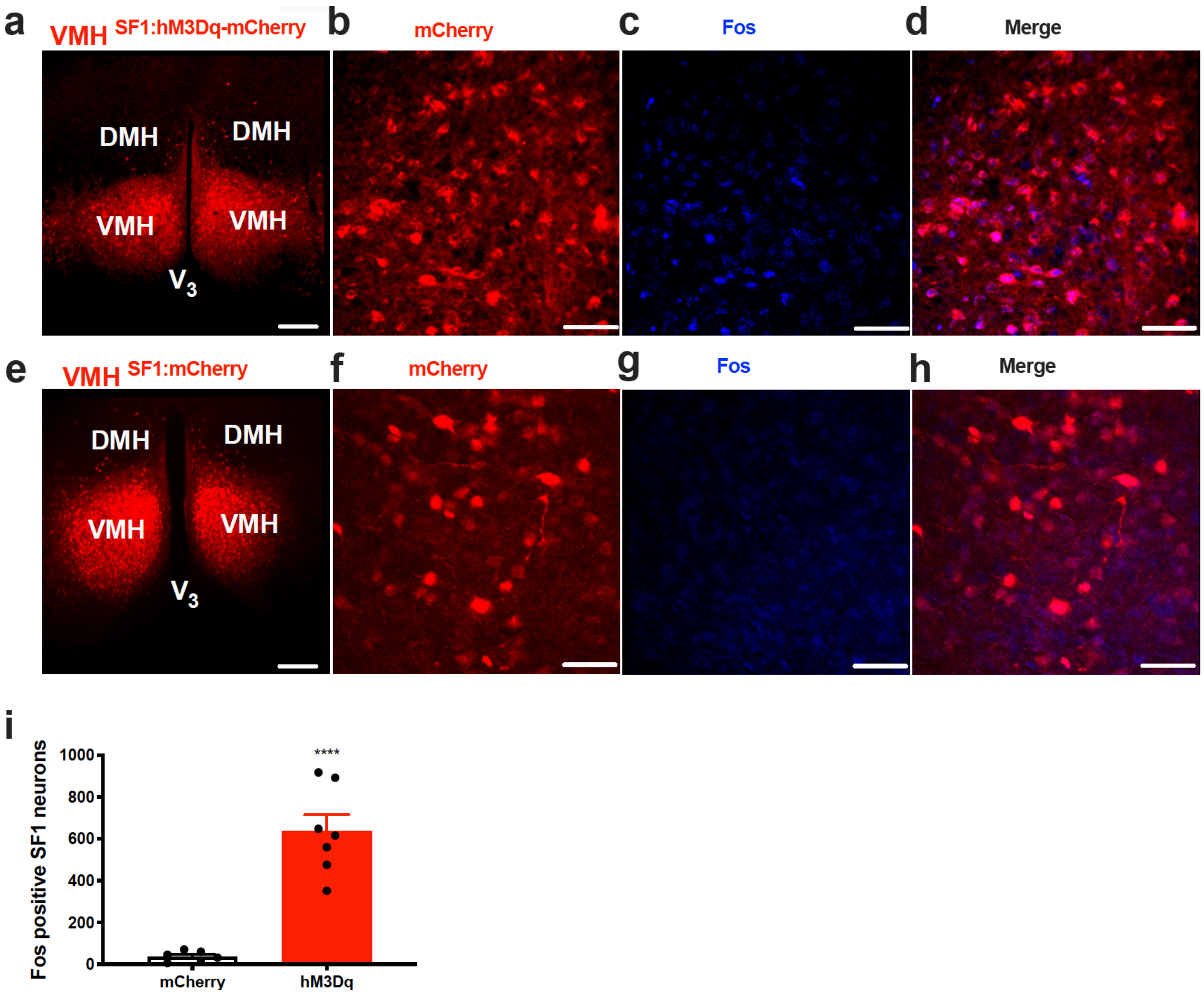

Supplementary Fig. 3. DREADD-based activation of VMH SF1 neurons. J60 i.p. injections potently increased Fos positive SF1 neurons in the SF1 neuron hM3Dq-mCherry transduced mice (7 sections/3 mice) as compared to mCherry-transduced mice (6 sections/3 mice). **a-d,** (**a**) Representative images of Cre-dependent expression of hM3Dq-mCherry in VMH SF1 neurons; (**b-d**) Sample images showing hM3Dq-mCherry (**b**; red), Fos (**c**; blue), and overlap of hM3Dq-mCherry with Fos (**d**). **e-h,** Representative image of Cre-dependent expressions of mCherry in VMH SF1 neurons (**e**); (**f-h**) Sample images showing hM3Dq-mCherry (**f**; red), Fos (**g**; blue), and overlap of hM3Dq-mCherry with Fos (**h**). **i,** Quantification of the percentage of Fos positive neurons co-expressing mCherry (mCherry, n=6; hM3Dq, n=7; p<0.0001). Scale bars, 200 µm for **a** and **e**; 50 µm for **b-d** and **f-h**. Two-tailed student t tests; data represent mean ± s.e.m; **** $p <$ 0.0001.

Supplementary Figure 4

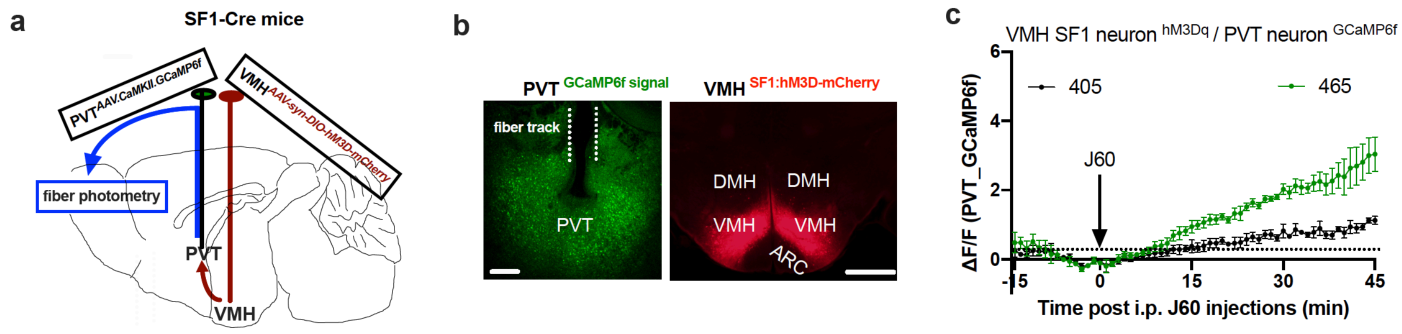

Supplementary Fig. 4. In vivo photometry monitoring of PVT neurons.

a, Schematic illustration of a viral infection and fiber photometry strategy. A vector carrying Cre-dependent hM3Dq-mCherry was targeted to the VMH to transduce SF1 neurons with hM3Dq-mCherry, and a second CaMKII-driven vector carrying $GCaMP_{6f}$ was injected into the PVT to transduce PVT neurons with $GCaMP_{6f}$ proteins in SF1-Cre mice. An optic fiber connected to the fiber photometry system was implanted on the surface of PVT to record $GCaMP_{6f}$ signals. J60 i.p. injections were administered to increase GCaMP6f signals. **b,** Sample images showing $GCaMP_{6f}$ signals in PVT (left) and hM3Dq-mCherry expression in VMH SF1 neurons (right). **c,** DREADD activation of VMH SF1 neurons through J60 i.p. injections enhanced $GCaMP_{6f}$ signals in PVT (n=4). The 465 nm and 405 nm signals were presented at 1-min bins. Data represent mean ± s.e.m. Scale bars, 200 μm for **b**. ARC, arcuate nucleus; DMH, dorsomedial hypothalamus; PVT, paraventricular hypothalamus; VMH, ventromedial hypothalamus.

Supplementary Figure 5

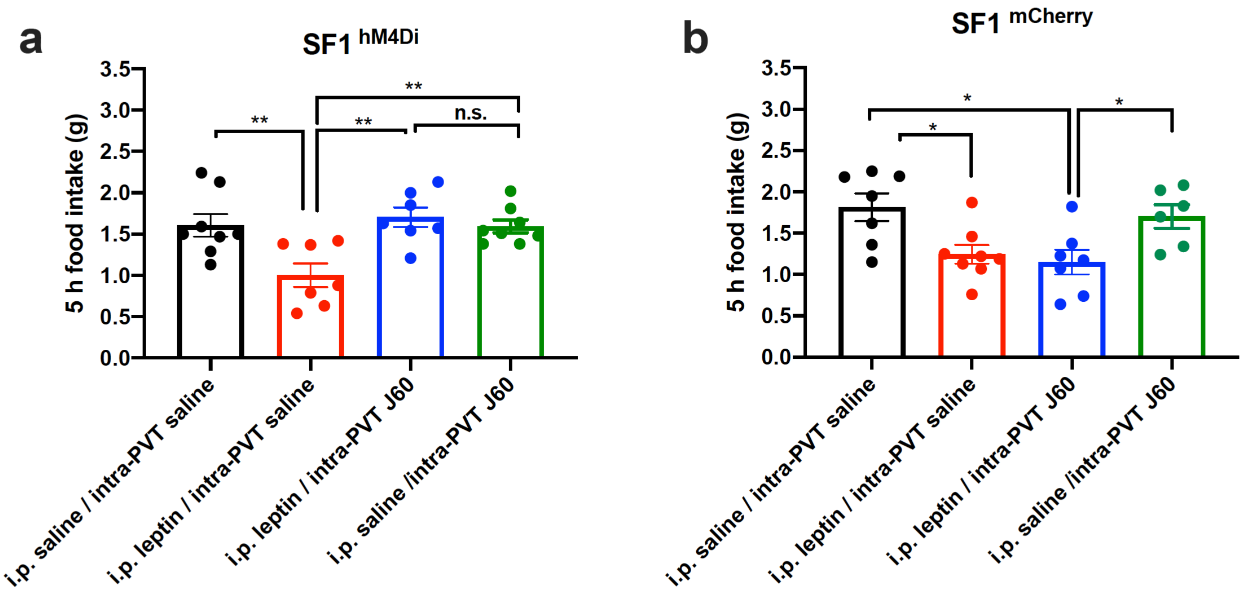

Supplementary Fig. 5. Chemogenetic inactivation of SF1 projections to PVT reduces leptin suppression of feeding.

Re-feeding after 15 h food deprivation was performed in the SF1 hM4Di or mCherry-transduced mice which were also implanted guide cannula in the PVT for intra-PVT injections of J60 or saline. **a**, Group data of 5-h food intake collected from the SF1 hM4Di-transduced mice simultaneously receiving i.p. saline and intra-PVT saline (n=8), i.p. leptin and intra-PVT saline (n=7), i.p. saline and intra-PVT J60 (n=8), or i.p. leptin and intra-PVT J60 (n=7) respectively (i.p saline /intra-PVT saline vs i.p leptin/intra-PVT saline, *p*=0.0079; i.p leptin/intra-PVT saline vs i.p leptin/intra-PVT J60, *p*=0.0026; i.p leptin/intra-PVT saline vs i.p saline/intra-PVT J60, *p*=0.0093, one-way ANOVA with Turkey *post hoc* tests). **b**, Group data of 5-h food intake collected from the SF1 mCherry-transduced mice simultaneously receiving i.p. saline and intra-PVT saline (n=7), i.p. leptin and intra-PVT saline (n=8), i.p. saline and intra-PVT J60 (n=6), or i.p. leptin and intra-PVT J60 (n=7) respectively [(i.p saline /intra-PVT saline vs i.p leptin/intra-PVT saline, *p*=0.0356; i.p saline/intra-PVT saline vs i.p leptin/intra-PVT J60, *p*=0.015; one-way ANOVA with Turkey *post hoc* tests); i.p. leptin/intra-PVT J60 vs i.p. saline/intra-PVT J60, *p*=0.0227, two-tailed student *t* test]. Data represent mean ± s.e.m; * $p < 0.05$; ** $p < 0.01$; n.s., not significant.

Supplementary Figure 6

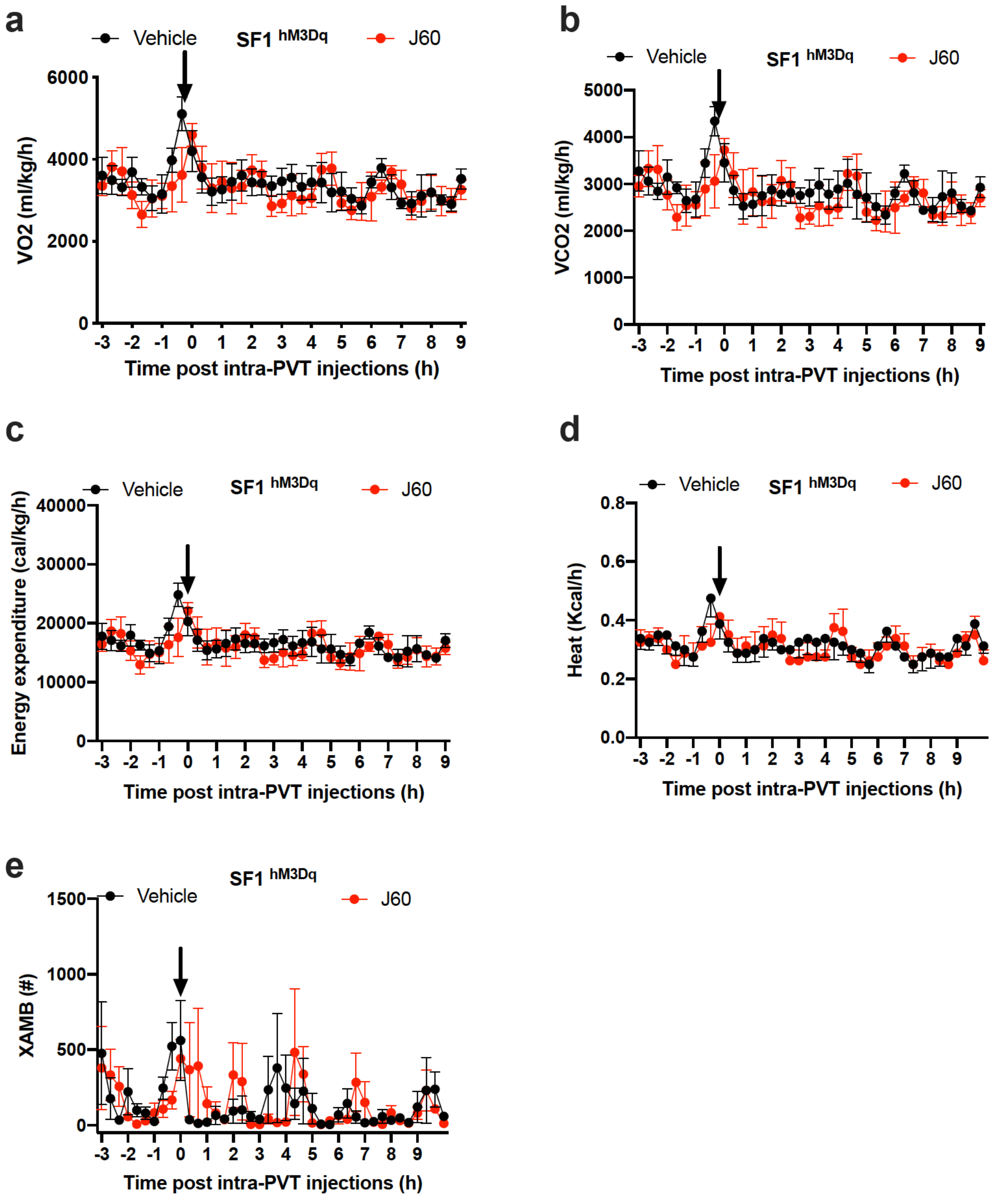

Supplementary Fig. 6. SF1 projections to PVT does not affect energy expenditure.

a-e, As compared to vehicle treatment, DREADD activation of SF1 projections to PVT with J60 via intra-PVT injections in SF1 hM3Dq-transduced mice (n=4) did not affect O_2 consumption (**a**), CO_2 production (**b**), energy expenditure (**c**), heat production (**d**), or locomotion (**e**). Data represent mean ± s.e.m.

Supplementary Figure 7

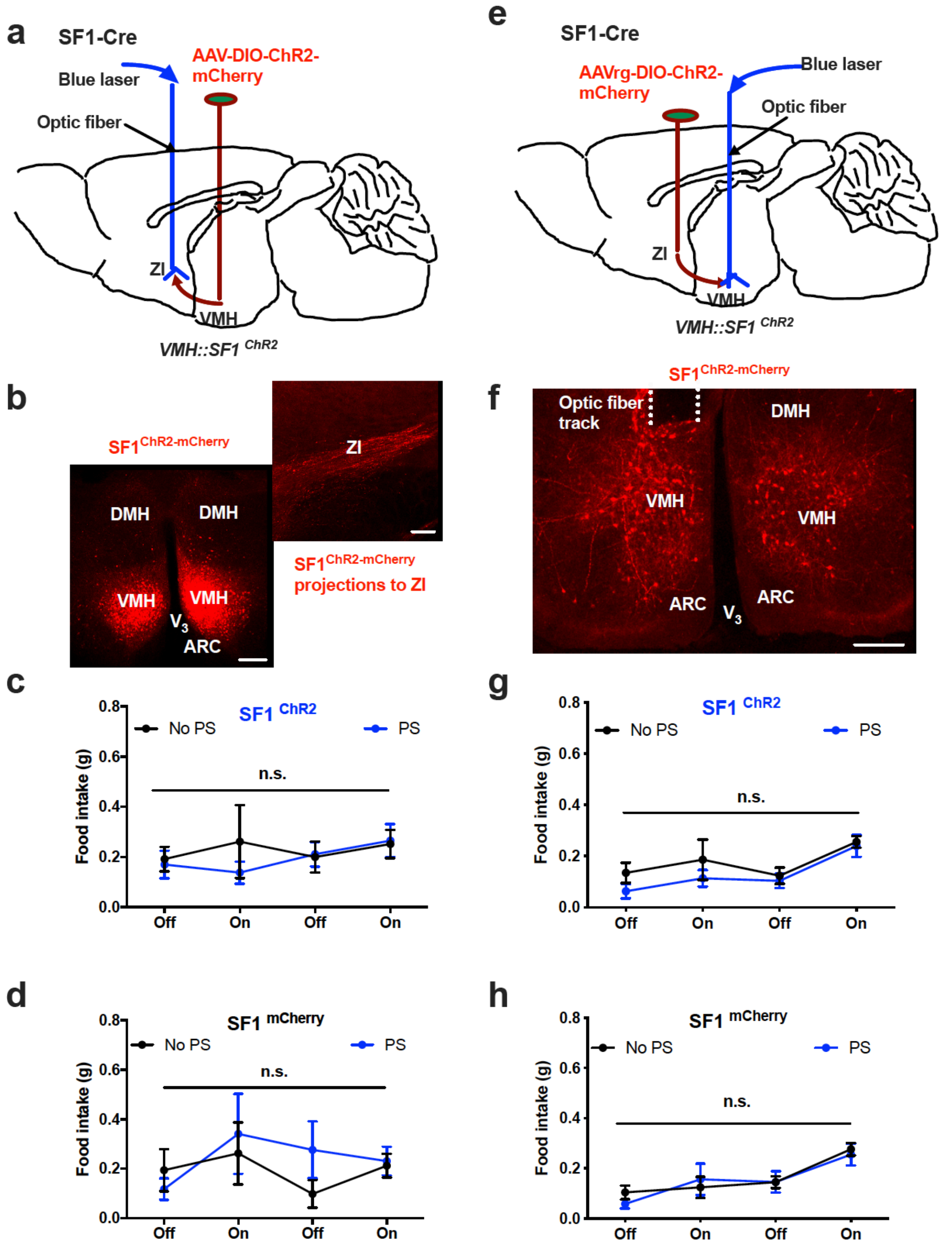

Supplementary Fig. 7. PS of SF1 projections to ZI or ZI-projecting SF1 neurons does not affect food intake. a-d, PS of SF1 projections to ZI did not affect feeding (n=6 per group): (**a**) Schematic illustration of transducing SF1 neurons in the VMH with ChR2-mCherry, and an optic fiber above ZI for PS in SF1-Cre mice; (**b**) Sample images of ChR2-mCherry transduced SF1 neurons in the VMH and their projections in the ZI; (**c**) Group data of food intake collected from the SF1 ChR2-transduced mice at the intervals of 30 min with blue laser off and on respectively; and (**d**) Group data of food intake collected from the SF1 mCherry-transduced mice. **e-h**, PS of ZI-projecting SF1 neurons did not affect food intake (n=6 per group): (**e**) An illustration of retrograde tracing and transducing ZI-projecting SF1 neurons with ChR2-mCherry, and an optic fiber above the VMH for PS in SF1-Cre mice; (**f**) Sample image of the retrograde traced ZI-projecting VMH SF1 neurons; (**g**) Group data of food intake collected from ChR2-transduced mice at the intervals of 30 min with blue laser off and on respectively; and (**h**) Group data of food intake collected from the mCherry-transduced mice. Two-way ANOVA with Sidak *post hoc* tests were performed. Data represent mean ± s.e.m. n.s., not significant. Scale bars, 200 μm. ARC, arcuate nucleus; DMH, dorsomedial hypothalamus; PS, photostimulation; V_3, third ventricle; VMH, ventromedial hypothalamus; ZI, zona incerta.

Supplementary Figure 8

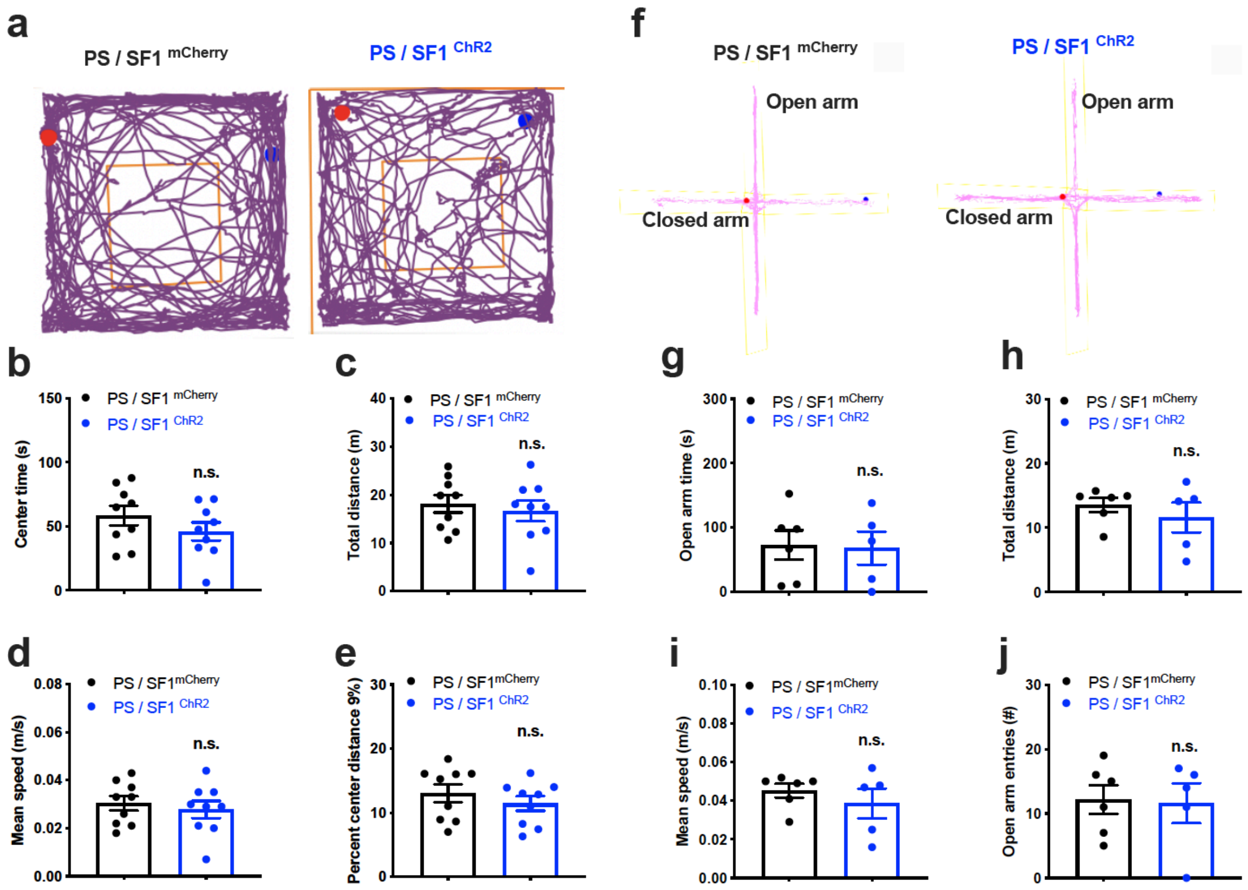

Supplementary Fig. 8. PS of SF1 projections to ZI does not elicit anxiety.

a-e, Open behavioral tests with PS of SF1 projections to ZI in the SF1 ChR2 or mCherry-transduced SF1-Cre mice: (**a**) Representative traces of open field behavioral tests in mice receiving PS 30 min before and during the tests in mCherry (left) and ChR2 (right)-transduced mice; (**b-e**) Group data of (**b**) time spent in the center of the open field (n=9 per group), (**c**) total distance traveled (n=9 per group), (**d**) mean speed (n=9 per group), and (**e**) percent center distance (n=9 per group). **f-j**, Elevated plus maze tests with PS of SF1 projections to ZI in SF1 ChR2 or mCherry-transduced mice were performed: (**f**) Representative traces of plus maze tests in mice receiving PS 30 min before and during the tests in mCherry (left) and ChR2 (right)-transduced mice; (**g-j**) Group data of (**g**) time spent in the open arms (mCherry, n=6; ChR2, n=5), (**h**) total distance traveled (mCherry, n=6; ChR2, n=5), (**i**) mean speed (mCherry, n=6; ChR2, n=5), and (**j**) open arm entry number (mCherry, n=6; ChR2, n=5). Two-tailed student t tests; data represent mean ± s.e.m. n.s. (not significant).

Supplementary Table 1

a LiCl conditioned flavor aversion protocol

Day 1-2	Day 3-4	Day 5	Day 6
Habituation (gel)	Test preference (initial preference)	Conditioning (2 sessions separated by 4 h; initially preferred gel following by LiCl and the other one by saline injection	Test preference (conditioned preference)

b SF1 neurons, SF1 projections to PVT and PVT neurons conditioned flavor aversion protocol

Day 1-2	Day 3-4	Day 5-8 (Conditioning X4)	Day 9-10
Habituation (gel)	Test preference (initial preference)	Conditioning (2 sessions separated by 4 h; initially preferred gel following by J60 and the other one by vehicle injection	Test preference (conditioned preference)

Supplementary Table 1. Experimental protocols and timeline for flavor aversion tests.
a, Timeline and protocol for LiCl conditioned flavor aversion. **b**, Timeline and protocol for flavor preference tests paired with DREADD manipulations of SF1 neurons, their projections and /or PVT neurons.
