# Supplementary material for: An excitatory ventromedial hypothalamus to paraventricular thalamus circuit that suppresses food intake
Source: Nat Commun. 2020 Dec 10;11:6326. doi: 10.1038/s41467-020-20093-4 (PMC7728757; doi:10.1038/s41467-020-20093-4)
Supplement: Supplementary file 3 — Reporting Summary [file 41467_2020_20093_MOESM3_ESM.pdf]

nature research

Corresponding author(s): Yang, Yunlei

Last updated by author(s): Nov 09, 2020

Reporting Summary

Nature Research wishes to improve the reproducibility of the work that we publish. This form provides structure for consistency and transparency in reporting. For further information on Nature Research policies, see Authors & Referees and the Editorial Policy Checklist .

Statistics

For all statistical analyses, confirm that the following items are present in the figure legend, table legend, main text, or Methods section.

n/a	Confirmed	
☐	☒	The exact sample size (*n*) for each experimental group/condition, given as a discrete number and unit of measurement
☐	☒	A statement on whether measurements were taken from distinct samples or whether the same sample was measured repeatedly
☐	☒	The statistical test(s) used AND whether they are one- or two-sided<br>*Only common tests should be described solely by name; describe more complex techniques in the Methods section.*
☒	☐	A description of all covariates tested
☐	☒	A description of any assumptions or corrections, such as tests of normality and adjustment for multiple comparisons
☐	☒	A full description of the statistical parameters including central tendency (e.g. means) or other basic estimates (e.g. regression coefficient) AND variation (e.g. standard deviation) or associated estimates of uncertainty (e.g. confidence intervals)
☐	☒	For null hypothesis testing, the test statistic (e.g. *F*, *t*, *r*) with confidence intervals, effect sizes, degrees of freedom and *P* value noted<br>*Give P values as exact values whenever suitable.*
☒	☐	For Bayesian analysis, information on the choice of priors and Markov chain Monte Carlo settings
☒	☐	For hierarchical and complex designs, identification of the appropriate level for tests and full reporting of outcomes
☒	☐	Estimates of effect sizes (e.g. Cohen's *d*, Pearson's *r*), indicating how they were calculated

Our web collection on statistics for biologists contains articles on many of the points above.

Software and code

Policy information about availability of computer code

Data collection	Fiber photometry data were collected using a two-channel dual-wavelength FP system (Doric Lenses); The images were taken using a Leica SP8 Confocal microscopy and/or an inverted microscopy (Olympus IX51); ChR2-assisted circuit mapping was performed by using an electrophysiology rig (Molecular Devices, MultiClamp 700B) quipped with photostimulation (CrystLaser 473 nm); Indirect calorimetry was performed using the Columbus Labs Comprehensive Lab Animal Monitoring Systems (CLAMS); Transgenic mice were genotyped using the Gel Documentation Systems (Corning). ANY-maze behavior tracking software 5.1 (Stoelting) was used to perform and analyze open field and plus maze behavioral tests.
Data analysis	All data were analyzed using Prism 7.0 (GraphPad Software). Student's t-tests were used to analyze differences between two groups of the same or different mice when appropriate, respectively. One-way ANOVA with post hoc test was used to compare group data from more than two groups of mice. Repeated measures (RM) two-way ANOVA with the within-subject factors of time segment and treatment (vehicle vs J60; PS off vs PS on) or mixed ANOVA with the within-subject factor of time segment and the between-subjects factor of viral injections type (control fluorescent proteins vs. hM3Dq) were used to analyze data from more than two groups across various time points. Sidak's post hoc test was used to test from significant effects at various time segments following the detection of a significant effects main effect or interaction. Fiber photometry data were analyzed using a software (Doric Studio V5.3.3.14). Electrophysiology data were analyzed using a software (pClampfit 10.7, Molecular Devices) as stated in the Method section.

For manuscripts utilizing custom algorithms or software that are central to the research but not yet described in published literature, software must be made available to editors/reviewers. We strongly encourage code deposition in a community repository (e.g. GitHub). See the Nature Research guidelines for submitting code & software for further information.

Data

Policy information about availability of data

All manuscripts must include a data availability statement. This statement should provide the following information, where applicable:

- Accession codes, unique identifiers, or web links for publicly available datasets
- A list of figures that have associated raw data
- A description of any restrictions on data availability

All the data generated and analyzed that support the findings in this study are available from the corresponding author upon reasonable request.

Field-specific reporting

Please select the one below that is the best fit for your research. If you are not sure, read the appropriate sections before making your selection.

[x] Life sciences   [ ] Behavioural & social sciences   [ ] Ecological, evolutionary & environmental sciences

For a reference copy of the document with all sections, see nature.com/documents/nr-reporting-summary-flat.pdf

Life sciences study design

All studies must disclose on these points even when the disclosure is negative.

Sample size	Our sample sizes are similar to those reported in previous publications (e.g. Betley JN et al 2013; Sweeney P et al 2015; 2017; Zhang X et al 2017).
Data exclusions	Following pre-established criteria (e.g. Sweeney P et al., 2015, 2017), mice with inaccurate viral infections or cannula placements were excluded from behavior experiments. Only mice with accurate viral injections and cannula placements were included in data analysis. Success rates for viral injections varied for each individual experiment with about 85% of injected animals displaying accurate viral injections and /or cannula placement. For the flavor preference test, mice which did not show initial preference were excluded for the next tests.
Replication	All experiments were repeated at least twice for each mouse and average values were calculated for each individual mouse for statistical analysis. All attempts at replication were successful.
Randomization	Male and female mice (half and half for each group unless where noted in the text and figure legends) were randomly assigned to experimental (DREADDs; ChR2) or control groups (control fluorescent protein) before viral injections and animal behavioral experiments.
Blinding	All behavioral experiments were performed and analyzed by an individual blind to the identification of pharmacological reagents (i.e. J60 vs vehicle) or vectors (ChR2 vs mCherry). Animal genotype was not performed in a blind manner as we need to screen transgenic-positive mice (e.g. Cre-expression mice).

Reporting for specific materials, systems and methods

We require information from authors about some types of materials, experimental systems and methods used in many studies. Here, indicate whether each material, system or method listed is relevant to your study. If you are not sure if a list item applies to your research, read the appropriate section before selecting a response.

Materials & experimental systems

n/a	Involved in the study	
[ ]	[x]	Antibodies
[x]	[ ]	Eukaryotic cell lines
[x]	[ ]	Palaeontology
[ ]	[x]	Animals and other organisms
[x]	[ ]	Human research participants
[x]	[ ]	Clinical data

Methods

n/a	Involved in the study	
[x]	[ ]	ChIP-seq
[x]	[ ]	Flow cytometry
[x]	[ ]	MRI-based neuroimaging

Antibodies

Antibodies used	Primary antibody anti-Fos (E-8) conjugated with Alexa fluoro 647 (sc-166940; Lot#A3020; Santa Cruz);<br>Primary antibody anti-vGluT2 (Invitrogen; MA5-27613);<br>Seconday goat anti-mouse IgG-conjugated with fluorophore Alexa fluoro 680 (Invitrogen; A21057).

Validation	The Fos antibody is reactive to mouse tissue, and has been cited in 63 publications, including Li R et al 2018; Xue Y et al 2019, which used this antibody in mouse brain tissues. In this study we used this antibody to stain Fos signals in the extended data Figure 3. We used the vGluT2 antibody to stain vGluT2 in the Figure 4, the detailed information of this antibody is provided on the manusfacture's website (https://www.thermofisher.com/antibody/product/VGLUT2-Antibody-clone-S29-29-Monoclonal/MA5-27613). The secondary goat-anti-mouse antibody Alexa 680 has been cited in 51 publications, including Wang P et al 2019.

Animals and other organisms

Policy information about studies involving animals; ARRIVE guidelines recommended for reporting animal research

Laboratory animals	Male wild-type C57BL/6J (Jax Stock No: 000664), SF1-Cre (Jax Stock No: 012462), and vGluT2-ires-Cre (Jax Stock No: 016963) have been described previously and purchased from The Jackson Laboratory. Transgenic mice were genotyped for Cre. Both male and female mice (age 5-8 weeks) were used at the start of experiments, unless otherwise noted. Mice were group-housed 3-5 mice per cage in temperature - and humidity-controlled rooms on a 12-h light:12-h dark cycle, with lights on from 8:00 a.m. to 8:00 p.m., and with ad libitum access to water and mouse regular chow (PicoLab Rodent Diet 20, 5058, LabDiet). Mice were single-caged after they received viral transductions with or without guide cannula insertion until all experimental procedures were finished. The virally transduced mice were randomly assigned to experimental and control groups at the start of experiments. Mice were also randomly assigned and evenly age and sex-matched for the different viral injections and treatments as described in the text and figure legends.
Wild animals	No wild animals were used in this study.
Field-collected samples	No field-collected samples were used in this study.
Ethics oversight	Experimental protocols were approved by the Institutional Animal Care and Use Committees at the Albert Einstein College of Medicine and conducted following the US National Institutes of Health guidelines for animal research.

Note that full information on the approval of the study protocol must also be provided in the manuscript.
